# Prevalence and associated risk factors for suicidal ideation, non-suicidal self-injury and suicide attempt among male construction workers in Ireland

**DOI:** 10.1186/s12889-024-18483-0

**Published:** 2024-05-08

**Authors:** Shane O’Donnell, Tom Egan, Nicholas Clarke, Noel Richardson

**Affiliations:** 1 National Centre for Men’s Health, South East Technological University (Carlow Campus), Kilkenny Road, Carlow, Ireland; 2 School of Business, South East Technological University (Waterford Campus), Cork Road, Waterford, Ireland; 3 RCSI University of Medicine and Health Sciences, 123 St Stephen’s Green, Dublin, Ireland

**Keywords:** Suicidal ideation, Non-suicidal self-injury, Suicide attempt, Construction industry, Male, Workplace

## Abstract

**Background:**

Suicide among male construction workers are reported to be disproportionally high compared to the working age population. However, there is minimal understanding of the prevalence and associated factors for suicidal ideation, non-suicidal self-injury, and suicide attempt among this occupational group globally.

**Methods:**

A cross-sectional study was conducted on a large sample of male construction workers in Ireland (*n* = 1,585). We investigated the prevalence of suicidal ideation, non-suicidal self-injury and suicide attempts and sociodemographic, occupational, and mental health factors associated with these three outcomes. Multivariable Poisson regression was performed to estimate the prevalence rate ratio of suicidal ideation (model 1 primary outcome), while multivariable logistic regression was used to estimate the odds ratio of non-suicidal self-injury (model 2 primary outcome), and suicide attempt (model 3 primary outcome).

**Results:**

The lifetime prevalence rate for suicidal ideation was 22%, 6% for non-suicidal self-injury, and 6% for suicide attempt. In univariate modelling, socio-demographic and occupation-specific factors associated with the three outcomes included younger age (suicidal ideation and non-suicidal self-injury), not being in a relationship (suicide attempt) and working 35–44 h per week (suicidal ideation and suicide attempt). The mental health factors generalized anxiety disorder, depression, and suicide bereavement were significantly associated with increased risk of the three outcomes. In fully adjusted multivariable models, increasing severity of generalized anxiety disorder and depression were associated with an increased prevalence rate ratio of suicidal ideation, and a higher odds ratio of non-suicidal self-injury and suicide attempt.

**Conclusion:**

Suicidal ideation, non-suicidal self-injury and suicide attempt are significant issues for male construction workers that require specific attention. Findings highlight a need to support younger male construction workers and those bereaved by suicide. They also highlight the need for the early detection and treatment of generalized anxiety disorder and depression in order to intervene in, and potentially prevent, suicidality among male construction workers.

**Supplementary Information:**

The online version contains supplementary material available at 10.1186/s12889-024-18483-0.

## Background

Suicide is a major public health concern and is estimated to account for 703,000 deaths per year and 1.3% of global deaths in 2019 [1]. Men are disproportionally affected by suicide where the global male suicide rate is approximately twice the female rate [1]. Therefore, it is perhaps not surprising that elevated suicide rates have been reported in the male-dominated construction industry, in countries such as the USA [2], the United Kingdom [3], Australia [4] and Finland [5]. Previous meta-analyses have indicited that construction workers have a 25–80% increased relative risk of suicide compared to the general working age population [6, 7].

The causes of suicide are complex and encompass a range of biological, psychological sociodemographic, environmental, and situational factors [8]. A ‘macho’ workplace culture and conformity to masculine norms– particularly self-reliance, risk-taking and emotional control - have been implicated in the relationship between construction workers and suicide [9–11]. Beyond this, a number of occupation-specific risk factors have been reported such as low job control, high work demands, job insecurity, the transient nature of work and long work hours, production pressure, workplace bullying, physical injury and chronic pain [12–16]. Other studies that have reported factors associated with construction industry worker suicide include young age, low education, financial, legal and relationship issues, low socioeconomic status, drug use, alcohol misuse, migration, and depression [14–16]. This emerging body of evidence has prompted the development and implementation of early intervention programmes within the construction industry which have shown promise in reducing sigma, improving suicide prevention literacy and improving intention to seek help and to offer help to colleagues [17, 18]. However, researchers are often limited in their ability to evaluate the direct and indirect effects of such early intervention programmes on suicide due to the relatively low base-rate behaviour and the subsequent requirement to have prohibitively large sample sizes [19] and because most variables of interest cannot be measured postmortem [8]. Therefore, researchers have suggested that understanding and mitigating the factors associated with the earlier stages of the suicidal trajectory– suicidal ideation, non-suicidal self-injury, and suicide attempt– as an alternative pragmatic approach [19] due to the association of these outcomes with future suicide deaths [20–22]. For the purpose of this study, suicidal ideation is defined as thoughts of engaging in behaviour intended to end one’s life [23]; non-suicidal self-injury (NSSI) refers to direct, deliberate destruction of one’s own body tissue without the intent to die [24]; and suicide attempt relates to engagement in potentially self-injurious behaviour in which there is at least some intent to die [23].

There is a dearth of global research exploring the prevalence and associated factors of suicidal ideation, non-suicidal self-injury and suicide attempt among male construction workers. Two studies have explored suicidal ideation among male construction workers and reported a twelve-month prevalence rate of 2.5% in the USA and a two week prevalence rate of 7.4% in Australia [9, 25]. An additional study on suicidal ideation among construction industry apprentices reported a twelve-month prevalence rate of 29.4% [26]. The risk factors most associated with suicidal ideation in these studies include depression, low earnings, high working hours, young age, contract type, drug/opioid use, alcohol abuse, and poor physical health [9, 25]. A further qualitative study also highlighted long-work hours, financial worries, relationship issues and suicide bereavement as key contributing factors [27]. While these studies are a useful starting point to understanding and mitigating the factors associated with suicidal ideation among male construction workers, no studies to date have explored the prevalence or associated factors of non-suicidal self-injury or suicide attempt among this occupational group despite calls for such research [26]. This is an important first step if more effective and tailored suicide prevention interventions are to be developed to address a wider range of suicidal trajectories. Moreover, there are a distinct lack of studies exploring the issue of suicide among male construction workers in Ireland, despite previous scholarly attention exploring suicide amongst different male and occupation groups perceived to face an increased risk of suicide [28–30]. This is surprising considering the increased risk of suicide among construction workers in high-income countries [6, 7]; that males account for 80% of suicides in Ireland [31]; and that the construction industry is the third largest employment sector for males in Ireland [32]. A recent study on probable deaths by suicide in Ireland found that male skilled-manual workers had the highest prevalence of probable deaths by suicide among decedents with available occupational data [33], while a psychological autopsy study of 133 suicides in Ireland also reported an overrepresentation of construction workers [34]. Furthermore, a recent report found that 87% of construction industry employers reported at least one incidence of employees being deemed unfit to work due their mental health state [35]. While these studies may indicate an elevated suicide risk among construction workers in Ireland, further research is needed. Therefore, the aim of this study was to explore the prevalence and risk factors for suicidal ideation, NSSI and suicide attempt among male construction workers in Ireland.

## Methods

### Study design

A cross-sectional study was conducted in the Irish construction industry between 1st March and 31st August 2022. Ethical approval was granted by the Southeast Technological University Carlow Ethics Committee (Reference No 391).

### Participants and data collection

There were approximately 151,800 males employed in the Irish construction industry at the time of data collection [32]. Individuals were eligible to participate if they identified as male, were ≥ 18 years old and worked on a construction site in Ireland. Convenience sampling methods were used to recruit participants via construction industry companies operating in Ireland. The most recent estimate for the number of construction industry companies operating in Ireland was 62,664, of which 99.7% were considered small and midsize enterprises (< 250 persons engaged) and just 0.03% were considered large enterprises (> 250 persons engaged) [36]. A representative body for the Irish construction industry sent a recruitment email to all 1,346 of its members. The lead researcher also circulated the recruitment email to the top 20 construction industry companies with the highest profit turnover. A total of 15 companies responded to the recruitment email sent by the representative body and the research team which represented a 1% response rate. Only one response was from the representative body email while the other 14 responses arose from the email sent directly by the research team. Of the 15 companies that responded: eight agreed to participate; four did not respond to a follow-up email; and an appropriate time could not be agreed with the remaining three. Six of these companies were small/medium enterprises and two were large enterprises. However, all participating companies were contractors who employed numerous other companies and/or subcontractors on their sites. Therefore, data collection was conducted among all construction workers present on-site rather than exclusively with the staff of the participating companies. Participating companies identified potential sites and relevant ‘gatekeepers’ to aid recruitment. Gatekeepers included environmental health and safety officers, occupational health nurses, medical staff and/or senior management. A video call was held with the gatekeepers to discuss the logistics of conducting the survey and effective recruitment approaches. Gatekeepers set a date for the survey and encouraged participation among staff via word of mouth, recruitment posters, information sheets and/or emails. All recruitment materials were provided by the research team. Participants were given at least seven days to decide if they would like to participate. Data collection time slots of 30 minutes were allocated to groups of 10–30 people based on occupation and/or spoken language. The data was collected on-site in canteens, meeting rooms and/or portacabins assigned for data collection. One meter spacing was provided between participants where possible to ensure privacy. The lead author provided an overview of the study, distributed study information sheets and obtained written informed consent before circulating the surveys. Participants returned the survey into a sealed box, were given a list of local mental health services and offered a €6 service station voucher for their time. Psychotherapists, mental health charity representatives, and/or on-site staff trained in mental health first aid and/or suicide intervention were present with the research team during data collection as an additional safety measure in the event that someone became distressed.

### Materials

The survey consisted of 19 questions and was available in six languages– English, Polish, Romanian, Portuguese, Latvian and Lithuanian. These languages were highlighted as the most widely spoken languages on-site by the participating construction companies. Translated and validated versions of the scales used in this study were accessed online for the six target languages (https://www.phqscreeners.com/). The remaining questions were translated by certified translators that specialized in health-related translation. The survey questions were then piloted with 25 construction workers who represented the six target languages to assess their understanding of the questions, the perceived relevance of the questions, and time to complete the survey (≈ 10 min). Construction workers reported the perceived negative impact of commute time and shift work that were missing from the pilot survey, and so these variables were included in the final survey. The variables included in the survey are outlined below and described in more detail in Additional File 1.

#### Suicidality outcomes

Three items were adapted from the Adult Psychiatric Morbidity Survey [37] to assess suicidal ideation (*yes/no*); NSSI (*yes/no*) and suicide attempt (*yes/no*) respectively. If participants answered yes, they were also asked when they last experienced these outcomes *(within the past year; more than 1 year ago*). These suicidality measures were also used to assess suicidality among a representative sample of the Irish population [38] and were taken from the Clinical Interview Schedule Revised [39]. The Clinical Interview Schedule Revised has demonstrated acceptable psychometric properties [40, 41].

#### Sociodemographic variables

Six sociodemographic items were included: age *(18–29; 30–49; 50+)*; country of birth *(Ireland; Europe and Great Britain; Rest of World)*; sexual orientation *(heterosexual; LGBTQI+)*; relationship status *(in a relationship; not in a relationship)*; educational level *(secondary school or below; trade qualification or diploma; tertiary)* and living alone *(yes/no)*.

#### Occupation-specific variables

Six occupation-specific items were included: occupation *(skilled trade, managerial role; unskilled labour)*; annual income *(≤€29,999; €30,000-€49,999; €50,000-€69,999; ≥€70,000)*; hours worked per week *(≤ 39 h; 40–44 h; 45–49 h; ≥50 h)*; contract *(permanent, not permanent);* shift work *(always, sometimes, never)*; and commute time *(≤ 1 h; >1 h)*.

#### Mental health variables

The Generalized Anxiety Disorder-7 (GAD-7) and Patient Health Questionnaire-9 (PHQ-9) were used to screen for general anxiety disorder (GAD) and depression [42, 43]. These validated scales ask participants to rank how often they have been bothered by a problem over the past two weeks on a four-point Likert scale (*0 = not at all; 3 = nearly every day*). Scores are summed and then categorized according to severity of generalized anxiety disorder (*GAD-7: 0–4 = minimal; 5–9 = mild; 10–14 = moderate; 15 + = severe*) and depression (*PHQ-9: 0–4 = minimal; 5–9 = mild; 10–14 = moderate; 15–19 = moderate/severe; 20 + = severe*). Finally, two items were added to assess financial worry (*measured on ten-point Likert scale and recoded into five categories– not at all worried; somewhat worried; worried; very worried; extremely worried*) and suicide bereavement *(yes/no).*

### Data analysis

All analysis was conducted in STATA 16. Characteristics of participants experiencing or not experiencing each lifetime suicidality outcome (suicidal ideation, NSSI and suicide attempt), were compared using chi-square tests and *t* tests as appropriate. Cronbach’s α was computed as a measure of internal reliability for the GAD-7 (α: 0.90) and PHQ 9 (α: 0.87), both of which were adequate. Given that the outcome for lifetime suicidal ideation was common (> 10%) logistic regression was not used for estimation [44]. Rather this outcome was modelled using Poisson regression with a log link and robust error variance [45] to estimate relative risks (RR). For the lifetime NSSI and suicide attempt outcomes, logistic regression was conducted to assess the associations between each outcome and the candidate sociodemographic, mental health, and occupation-specific variables. To develop these models, associations were initially investigated for each outcome in three blocks: [1] each lifetime suicidality outcome and sociodemographic variables; [2] each lifetime suicidality outcome and occupation-specific variables; [3] and each lifetime suicidality outcome and mental health variables. The GAD-7 and PHQ-9 severity categories were used rather than the cutoff point of ≥ 10. For each lifetime suicidality outcome, variables which were statistically significant in univariate models (Wald test for Poisson regression model and likelihood ratio test for logistic regression models) in each block, were fitted together. Then, for each outcome separately, all significant variables from the three blocks were fitted together in the final three models. Interactions between the socio-demographic variables and other variables in the model were checked. Each of the three final models had adequate fit, based on the Hosmer & Lemeshow test. Variables in the three final models had variance inflation factors of < 10 and tolerance > 0.1 [46]. A test of trend was used to assess linear trends in outcomes within continuous variables. Univariate and multivariate analyses on the 12-month suicidality outcome was not possible due to numbers being below the recommended amount for logistic regression analyses.

## Results

A total of 1,585 male construction workers completed the survey in 103 separate time slots across 52 construction sites. These sites were geographically spread across three of the four provinces of Ireland (Leinster 53.8%; Munster 26.9%; Connacht 19.2%). Participating construction companies estimated a workforce of 8,045 across the 52 sites giving an approximate sample response rate of 19.7% or 1% of the total male workforce in the Irish construction industry. The mean age of the sample was 35 years old (IQR 25–43), 81% were born in Ireland, 97.9% were heterosexual, 71.7% were in a relationship, 9.3% lived alone, 36.7% had completed no more than primary or secondary education, 21.8% had an annual yearly salary of ≤€29,999; and 67.5% had a permanent contract. A total of 13.3% and 12.9% screened positive for depression and GAD respectively (score of ≥ 10) and 46.1% of participants were bereaved by suicide. A detailed description of overall participant characteristics is included in Additional File 2. Participants characteristics as they relate to suicidal ideation, NSSI and suicide attempt are provided in Table 1 with p values that represent differences within the examined variables across the suicidality outcomes.

**Table 1:** Participant characteristics by lifetime suicidal ideation, NSSI and suicide attempt with associated p values

	Lifetime suicidal ideation					Lifetime NSSI					Lifetime suicide attempt				
	Yes		No		p value	Yes		No		p value	Yes		No		p value
	N	%	N	%		N	%	N	%		N	%	N	%	
**Prevalence rate**															
	346	22.4	1,197	77.6	-	93	6.0	1,450	94.0	-	94	6.1	1,449	93.9	-
**Age**															
18–29	152	25.8	437	74.2	***0.038***	50	8.5	539	91.5	***0.002***	44	7.5	545	92.5	0.200
30–49	157	21.2	584	78.8		37	5.0	704	95.0		39	5.3	702	94.7	
50+	36	18.2	162	81.8		5	2.5	193	97.5		10	5.1	188	95.0	
**Region of birth**															
Ireland	290	23.3	956	76.7	0.160	78	6.3	1,168	93.7	0.520	80	6.4	1,166	93.6	0.356
Europe & Great Britain	41	17.7	191	82.3		11	4.7	221	95.3		10	4.3	222	95.7	
Rest of world	11	20.4	43	79.6		2	3.7	52	96.3		2	3.7	52	96.3	
**Sexual orientation**															
Heterosexual	337	22.5	1,159	77.5	0.816	89	6.0	1,407	94.1	0.453	92	6.2	1,404	93.9	0.458
LGBTQ+	8	24.2	25	75.8		3	9.1	30	90.9		1	3.0	32	97.0	
**Relationship status**															
In relationship	234	21.1	873	78.9	0.060	63	5.7	1,044	94.3	0.365	57	5.2	1,050	94.9	***0.013***
Not in relationship	111	25.6	323	74.4		30	6.9	404	93.1		37	8.5	397	91.5	
**Living alone**															
No	310	22.2	1,086	77.8	0.344	81	5.8	1,315	94.2	0.190	83	6.0	1,313	94.1	0.368
Yes	36	25.7	104	74.3		12	8.6	128	91.4		11	7.9	129	92.1	
**Education**															
Primary/secondary	127	22.4	441	77.6	0.940	40	7.0	528	93.0	0.345	42	7.4	526	92.6	0.224
Trade/diploma	152	22.1	535	77.9		35	5.1	652	94.9		37	5.4	650	94.6	
Tertiary	66	23.2	219	76.8		18	6.3	267	93.7		14	4.9	271	95.1	
**Contract type**															
Permanent	234	22.7	796	77.3	0.622	62	6.0	968	94.0	0.988	64	6.2	966	93.8	0.870
Not permanent	108	21.6	392	78.4		30	6.0	470	94.0		30	6.0	470	94.0	
€29,999 or less	76	23.2	251	76.8	0.981	23	7.0	304	93.0	0.336	22	6.7	305	93.3	0.751
€30,000-€49,999	138	22.6	473	77.4		40	6.6	571	93.5		38	6.2	573	93.8	
€50,000-€69,999	85	22.0	302	78.0		16	4.1	371	95.9		19	4.9	368	95.1	
€70,000 or more	39	22.2	137	77.8		10	5.7	166	94.3		10	5.7	166	94.3	
**Occupation**															
Skilled trade	196	21.2	728	78.8	0.094	60	6.5	864	93.5	0.756	55	6.0	869	94.1	0.847
Managerial	74	27.5	195	72.5		16	6.0	253	94.1		18	6.7	251	93.3	
Unskilled labour	65	23.0	218	77.0		15	5.3	268	94.7		19	6.7	264	93.3	
**Shift work**															
Always	50	24.0	158	76.0	0.372	11	5.3	197	94.7	0.768	16	7.7	192	92.3	0.393
Sometimes/seldom	103	24.4	319	75.6		28	6.6	394	93.4		29	6.9	393	93.1	
Never	189	21.2	701	78.8		52	5.8	838	94.2		49	5.5	841	94.5	
**Hours worked per week**															
< 35 h worked per week	61	17.2	293	82.8	**0.020**	20	5.7	334	94.3	0.738	13	3.7	341	96.3	0.053
35–44 h worked per week	227	24.5	699	75.5		54	5.8	872	94.2		67	7.2	859	92.8	
45 + hours worked per week	57	22.3	199	77.7		18	7.0	238	93.0		14	5.5	242	94.5	
**Commute time**															
≤ 1 h	215	22.2	754	77.8	0.718	55	5.9	912	94.1	0.730	55	5.7	914	94.3	0.356
> 1 h	131	23.0	439	77.0		36	6.3	534	93.7		39	6.8	531	93.2	
**Suicide bereavement**															
No	146	17.6	686	82.5	***< 0.001***	37	4.5	795	95.6	***0.005***	32	3.9	800	96.2	***< 0.001***
Yes	200	28.1	511	71.9		56	7.9	655	92.1		62	8.7	649	91.3	
**Financial worry**															
Not at all worried	103	17.4	489	82.6	***< 0.001***	26	4.4	566	95.6	***0.006***	27	4.6	565	95.4	***0.006***
Somewhat worried	56	18.8	242	81.2		14	4.7	284	95.3		12	4.0	286	96.0	
Worried	91	24.5	280	75.5		26	7.0	345	93.0		26	7.0	345	93.0	
Very worried	61	31.8	131	68.2		14	7.3	178	92.7		19	9.9	173	90.1	
Extremely worried	34	39.5	52	60.5		12	14.0	74	86.1		10	11.6	76	88.4	
**GAD-7 category**															
Minimal GAD	102	10.5	869	89.5	***< 0.001***	22	2.3	949	97.7	***< 0.001***	20	2.1	951	97.9	***< 0.001***
Mild GAD	123	33.5	244	66.5		29	7.9	338	92.1		28	7.6	339	92.4	
Moderate GAD	83	55.7	66	44.3		29	19.5	120	80.5		31	20.8	118	79.2	
Severe GAD	36	70.6	15	29.4		12	23.5	39	76.5		14	27.5	37	72.6	
**PHQ-9 category**															
No depression	107	11.0	864	89.0	***< 0.001***	21	2.2	950	97.8	***< 0.001***	14	1.4	957	98.6	***< 0.001***
Mild depression	116	32.0	246	68.0		39	10.8	323	89.2		35	9.7	327	90.3	
Moderate depression	73	53.3	64	46.7		12	8.8	125	91.2		23	16.8	114	83.2	
Moderate/severe depression	34	66.7	17	33.3		15	29.4	36	70.6		15	29.4	36	70.6	
Severe depression	16	88.9	2	11.1		6	33.3	12	66.7		7	38.9	11	61.1	

### Prevalence of suicidal ideation, NSSI and suicide attempt

Male construction workers in Ireland reported lifetime prevalence rates of 22.4% for suicidal ideation, 6% for NSSI and 6.1% for suicide attempt. The twelve-month prevalence rates were 10.2% for suicidal ideation, 1.1% for NSSI and 1% for suicide attempt respectively. There were significantly higher rates of lifetime suicidal ideation among men who: were 18–29 year olds (26%; *P* = 0.038); worked 35–44 h per week (60%; *P* = 0.020); had very high or extreme levels of financial worry (32% and 40%; *p* < 0.001); were bereaved by suicide (28%; *p* < 0.001); had moderate or severe GAD (56% and 71%; *p* < 0.001); and had moderate/severe or severe depression (67% and 89%; *p* < 0.001). There was a significantly higher rate of lifetime NSSI among men who: were 18–29-year-olds (9%; *p* = 0.002); were bereaved by suicide (8%; *P* = 0.005); had extreme levels of financial worry (14%; *p* = 0.006); had severe GAD (24%; *p* < 0.001) and had severe depression (33%; *p* < 0.001). Finally, there was a significantly higher rate of lifetime suicide attempt among men who: were not in a relationship (9%; *p* = 0.013); were bereaved by suicide (9%; *p* < 0.001); had extreme levels of financial worry (12%; *P* = 0.006); had severe GAD (28%; *p* < 0.001) and had severe depression (39%; *P* < 0.001).

### Factors associated with lifetime suicidal ideation, non-suicidal self-injury and suicide attempt

#### Univariate models

Younger age, working 35–44 h per week, increasing levels of financial worry, increasing severity of GAD and increasing severity of depression were associated with lifetime suicidal ideation in the univariate models (Table 2). Younger age, suicide bereavement, increasing levels of financial worry, increasing severity of GAD and increasing severity of depression were associated with lifetime NSSI in the univariate models (Table 2). Finally, not being in a relationship, working 35–44 h per week, suicide bereavement, increasing levels of financial worry, increasing severity of GAD and increasing severity of depression were associated with lifetime suicide attempt in the univariate models (Table 2).

**Table 2:** Univariate models by sociodemographic, occupation-specific, and mental health variables for lifetime suicidal ideation, NSSI and suicide attempt

	Lifetime suicidal ideation			Lifetime NSSI			Lifetime suicide attempt		
	IRR^a^	95% CI	Wald test	OR^b^	95% CI	LRT^c^	OR	95% CI	LRT
**Block 1 Sociodemographic variables**									
**Age**									
18–29	Ref	-	***0.012***	Ref	-	***< 0.001***	Ref	-	0.099
30–49	0.82	0.68–1.00		0.57	0.37–0.88		0.69	0.44–1.07	
50+	0.70	0.51–0.98		0.28	0.11–0.71		0.66	0.33–1.34	
*Trend*	0.83	0.72–0.96		0.55	0.39–0.78		0.76	0.55–1.06	
**Education**									
Primary/secondary	Ref	-	0.840	Ref	-	0.465	Ref	-	0.103
Trade/diploma	0.99	0.80–1.22		0.71	1.44–1.13		0.71	0.45–1.13	
Tertiary	1.03	0.80–1.34		0.89	0.5–1.58		0.65	0.35–1.21	
*Trend*	1.01	0.89–1.15		0.90	0.67–1.2		0.78	0.58–1.05	
**Country of Birth**									
Ireland	Ref	-	0.484	Ref	-	0.339	Ref	-	0.288
Europe & GB	0.76	0.57–1.02		0.75	0.39–1.42		0.66	0.33–1.29	
Rest of world	0.88	0.51–1.50		0.58	0.14–2.41		0.56	0.13–2.34	
**Sexual orientation**									
Heterosexual	Ref	-	0.814	Ref	-	0.483	Ref	-	0.415
LGBTQ+	1.08	0.58–1.98		1.58	0.47–5.28		0.48	0.06–3.53	
**Relationship status**									
In relationship	Ref	-	0.058	Ref	-	0.371	Ref	-	***0.016***
Not in relationship	1.21	0.99–1.47		1.23	0.78–1.93		1.72	1.12–2.64	
**Living alone**									
Yes	Ref	-	0.335	Ref	-	0.213	Ref	-	0.386
No	0.86	0.64–1.16		0.66	0.35–1.24		0.74	0.39–1.43	
**Block 2 Occupation-specific variables**									
**Contract type**									
Permanent	Ref	-	0.623	Ref	-	0.988	Ref	-	0.870
Not permanent	0.95	0.78–1.16		1.00	0.64–1.56		0.96	0.62–1.51	
**Annual salary**									
€29,999 or less	Ref	-	0.703	Ref	-	0.177	Ref	-	0.382
€30,000-€49,999	0.97	0.76–1.24		0.93	0.54–1.58		0.92	0.53–1.58	
€50,000-€69,999	0.95	0.72–1.24		0.57	0.3–1.1		0.72	0.38–1.35	
€70,000 or more	0.95	0.68–1.34		0.80	0.37–1.71		0.84	0.39–1.81	
*Trend*	0.98	0.89–1.09		0.85	0.67–1.08		0.90	0.71–1.14	
**Occupation**									
Skilled trade	Ref		0.087	Ref		0.750	Ref		0.848
Managerial	1.30	1.03–1.63		0.91	0.52–1.61		1.13	0.65–1.96	
Unskilled labour	1.08	0.85–1.39		0.81	0.45–1.44		1.14	0.66–1.95	
**Shift work**									
Always	Ref	-	0.180	Ref	-	0.919	Ref	-	0.177
Sometimes/ Seldom	1.02	0.76–1.36		1.27	0.62–2.61		0.89	0.47–1.67	
Never	0.88	0.67–1.16		1.11	0.57–2.17		0.70	0.39–1.26	
*Trend*	0.95	0.88–1.02		0.99	0.83–1.18		0.89	0.75–1.05	
**Hours worked per week (hpw)**									
< 35 hpw	Ref		***0.025***	Ref		0.747	Ref		***0.041***
35–44 hpw	1.42	1.10–1.84		1.03	0.61–1.75		2.05	1.11–3.75	
45 + hpw	1.29	0.94–1.79		1.26	0.65–2.44		1.52	0.70–3.29	
**Block 3 Mental health variables**									
**Suicide bereavement**									
No	Ref	-	***< 0.001***	Ref	-		Ref	-	
Yes	1.60	1.33–1.94		1.84	1.2–2.82	***0.005***	2.39	1.54–3.70	***< 0.001***
**Financial worry**									
Not at all worried	Ref	-	***< 0.001***	Ref	-	***0.001***	Ref	-	***< 0.001***
Somewhat worried	1.08	0.8–1.45		1.07	0.55–2.09		0.88	0.44–1.76	
Worried	1.41	1.1–1.81		1.64	0.94–2.87		1.58	0.91–2.75	
Very worried	1.83	1.39–2.4		1.71	0.88–3.35		2.30	1.25–4.23	
Extremely worried	2.27	1.66–3.11		3.53	1.71–7.29		2.75	1.28–5.91	
*Trend*	1.23	1.15–1.32		1.31	1.12–1.54		1.33	1.13–1.56	
**GAD-7 category**									
Minimal GAD	Ref	-	***< 0.001***	Ref	-	***< 0.001***	Ref	-	***< 0.001***
Mild GAD	3.19	2.53–4.03		3.70	2.1–6.53		3.93	2.18–7.06	
Moderate GAD	5.30	4.2–6.69		10.42	5.8–18.73		12.49	6.90–22.62	
Severe GAD	6.72	5.21–8.67		13.27	6.13–28.75		17.99	8.43–38.39	
*Trend*	1.98	1.85–2.13		2.60	2.11–3.21		2.85	2.30–3.51	
**PHQ-9 category**									
No depression	Ref	-	***< 0.001***	Ref	-	***< 0.001***	Ref	-	***< 0.001***
Mild depression	2.91	2.3–3.67		5.46	3.17–9.42		7.32	3.89–13.77	
Moderate depression	4.84	3.81–6.13		4.34	2.09–9.04		13.79	6.90–27.56	
Mod/severe depression	6.05	4.65–7.88		18.85	8.98–39.56		28.48	12.79–63.44	
Severe depression	8.07	6.33–10.28		22.62	7.75–66.02		43.50	14.7–128.69	
*Trend*	1.79	1.69–1.89		2.23	1.86–2.67		2.66	2.22–3.20	

^a^PRR= Prevalence Rate Ratio; ^b^OR=Odds Ratio; ^c^LRT = Likelihood ratio test

#### Multivariate models

##### Suicidal ideation

GAD and depression were associated with lifetime suicidal ideation in the fully adjusted multivariate model. For each unit increase on the GAD-7, the risk of suicidal ideation increased by 30% (Trend: IRR 1.30, 95% CI 1.17–1.44; *p* < 0.001). Those with severe GAD had twice the risk of experiencing suicidal ideation (IRR 2.00, 95% CI 1.44–2.78) compared to those with minimal GAD. For each unit increase in the PHQ-9, the risk of lifetime suicidal ideation increased by 16% (Trend: IRR 1.16, 95% CI 1.06–1.27, *p* < 0.001). Those with severe depression had a 90% higher risk of experiencing suicidal ideation (IRR 1.90, 95% CI 1.37–2.64) compared to those with no depression (Table 3).

**Table 3:** Multivariable models for lifetime suicidal ideation, NSSI and suicide attempt (Wald and Likelihood ratio test)

			Lifetime suicidal ideation^a^			Lifetime NSSI^b^			Lifetime suicide attempt^c^		
	Yes N(%)	No N(%)	IRR^d^	95% CI	Wald test	OR^e^	95% CI	LRT^f^	OR	95% CI	LRT
**GAD-7 category**											
Minimal GAD	106 (10.5%)	902 (89.5%)	1.00	-	***< 0.001***	1.00	-	***< 0.001***	1.00	-	***0.002***
Mild GAD	131 (32.8%)	268 (67.2%)	1.53	1.19–1.97		2.16	1.12–4.17		1.54	0.78–3.05	
Moderate GAD	85 (54.5%)	71 (45.5%)	1.89	1.43–2.49		5.38	2.53–11.43		3.23	1.50–6.95	
Severe GAD	44 (71.0%)	18 (29.0%)	2.00	1.44–2.78		4.68	1.70-12.89		2.94	1.09–7.90	
*Trend*	-	-	1.30	1.17–1.44		1.89	1.42–2.61		1.65	1.51–2.57	
**PHQ-9 category**											
No depression	110 (10.9%)	901 (89.1%)	1.00	-	***< 0.001***	1.00	-	***0.006***	1.00	-	***< 0.001***
Mild depression	120 (31.2%)	265 (68.8%)	1.34	1.05–1.72		3.11	1.64–5.90		5.15	2.50-10.61	
Moderate depression	80 (53.0%)	71 (47.0%)	1.49	1.11–1.99		1.48	0.61–3.60		6.37	2.67–15.21	
Moderate/ severe depression	39 (67.2%)	19 (32.8%)	1.58	1.15–2.18		5.38	2.09–13.79		11.75	4.30-32.12	
Severe depression	19 (90.5%)	2 (9.5%)	1.90	1.37–2.64		5.07	1.34–19.12		18.79	5.09–69.39	
Trend	-	-	1.16	1.06–1.27		1.46	1.11–1.90		1.97	1.21–2.25	

^a^Poisson regression model as the outcome (suicidal ideation) was common (> 10%), model fully adjusted for all variables in blocks 1,2 & 3; ^b^Logistic regression: models fully adjusted for all variables in blocks 1,2 & 3;^c^PRR= Prevalence Rate Ratio; ^d^OR=Odds Ratio;^e^LRT = Likelihood Ratio Test

##### NSSI

GAD and depression were associated with lifetime NSSI in the fully adjusted multivariate model. For each unit increase on the GAD-7 there was an 93% increase in the odds of experiencing NSSI (Trend: OR 1.93, 95% CI 1.42–2.61; *p* < 0.001). Those with severe GAD had 4.7 times the odds of experiencing NSSI (OR 4.68, 95% CI 1.70–12.89) compared to than those with minimal GAD (Table 3). For each unit increase in the PHQ-9, the odds of NSSI increased by 46% (Trend: OR 1.46, 95% CI 1.11–1.90, *p* = 0.006). Those with severe depression had 5.1 times the odds of experiencing NSSI (Trend: OR 5.07, 95% CI 1.34–19.12) compared to those with no depression (Table 3).

##### Suicide attempt

GAD and depression were associated with lifetime suicide attempt in the fully adjusted model. For each unit increase on the GAD-7 there was an 65% increase in the odds of experiencing a suicide attempt (Trend: OR 1.65, 95% CI 1.51–2.57; *p* < 0.002). Those with severe GAD had 2.9 times the odds of experiencing a suicide attempt (OR 2.94, 95% CI 1.09–7.90) compared to than those with minimal GAD (Table 3). For each unit increase in the PHQ-9, the odds of a suicide attempt increased by 97% (Trend: OR 1.97, 95% CI 1.21–2.25, *P* < 0.001). Those with severe depression had 18.8 times the odds of experiencing a suicide attempt (OR 18.79, 95% CI 5.09–69.39) compared to those with no depression (Table 3).

## Discussion

Male construction workers in Ireland reported lifetime prevalence rates of 22.4% for suicidal ideation, 6% for NSSI and 6.1% for suicide attempt. The twelve-month prevalence rates were 10.2% for suicidal ideation, 1.1% for NSSI and 1% for suicide attempt respectively. All lifetime suicidality outcomes are lower than those reported among a national representative sample of the Irish population using the same measures during COVID-19 [38]. This may suggest that construction workers in Ireland do not have elevated lifetime rates of suicidal ideation, NSSI or suicide attempt. This finding is consistent with previous studies that report lower suicidal ideation among male construction workers compared to males working in other industries [9, 25] but deviates from previous meta-analyses that suggest male construction workers face an increased risk of death by suicide compared to the general population [6, 7]. While Tyler et al. postulated that differences in later suicidal trajectories (e.g. NSSI and suicide attempt) might explain this disparity [9], the findings of this study reported lower rates of NSSI and suicide attempt among construction workers compared to the general population. Previous research has suggested that the gender paradox of suicide– men’s higher rates of suicide but lower rates of suicidal ideation and suicide attempt compared to females– might be partially explained by a conformity to masculine norms that contributes to an underreporting of suicidal experiences among men and/or use of more lethal suicide means [47]. Considering that male construction workers display higher conformity to masculine norms compared to men in other industries [37], perhaps this disparity in suicide deaths and suicidal ideation, NSSI and suicide attempt between construction workers and the general population could also be partially explained by an underreporting of suicidal experiences and/or use of more lethal suicide means.

In relation to previous studies in the construction industry, suicidal ideation rates among male construction workers in Ireland are higher compared to their international counterparts [9, 25]. Although item-nine of the PHQ-9 was not the primary outcome measure for suicidal ideation in this study, when using this item as a measure of current suicidal ideation in this study as per Tyler et al. [9], the Irish rate was higher than the Australian rate (9.3% vs. 7.4%). The twelve-month suicidal ideation prevalence rate in Ireland was also higher than the 2.5% reported in the USA [25]. While no comparative data is available for NSSI and suicide attempt in the international construction industry, the lifetime and twelve-month prevalence rates for suicide attempt are higher than global estimates in high-income countries [21] while the NSSI rates are somewhat similar [48]. While the use of different outcome measures to assess suicidality challenges robust comparison between these populations, it is still apparent that suicidal ideation, NSSI and suicide attempt are significant issues in the Irish construction industry that requires early intervention to mitigate the factors associated with these suicidal trajectories.

This study highlighted a number of variables that were associated with different suicidal trajectories that could be leveraged in suicide prevention strategies among construction workers in Ireland and more broadly. Younger age was associated with suicidal ideation and NSSI in univariate models. While this is consistent with international studies that have highlighted the vulnerability of young male construction workers and apprentices to suicidal ideation [25, 26], it differs from national studies that report a higher prevalence of suicidal ideation and deaths by suicide among older males [31, 37, 49]. Previous research suggests that a problematic culture of workplace bullying and substance may be explanatory factors for the high rates of suicidal ideation among young male construction workers [26, 50]. Moreover, self-reliance has been reported to have a particularly strong relationship with suicidal ideation among young males [51] and construction workers [9] whereby remaining stoic and not seeking help compounds suicidal ideation when distress is high [52]. Considering that young males are among the least likely to be engaged with mental health services when experiencing mental ill-health and/or suicidal ideation [49, 53], early intervention workplace initiatives that reframe self-reliance and encourage help-seeking are particularly needed for this occupational group. Indeed, a recent study in Ireland examining the clinical and demographic risk profiles of suicide cases identified young males as a priority group to target in occupational settings [54]. Not being in a relationship was also associated with an increased risk of suicide attempt, one of the foremost risk factors for male suicide attempts and death by suicide [55]. It has been postulated that norms of male emotional suppression may impact men’s interpersonal relationships and lead to an overreliance on intimate partners for emotional support [56]. Therefore, the absence or loss of this support may compound distress and result in a transition from suicidal ideation to a suicide attempt among this occupational group. Finally, suicide bereavement was also associated with suicidal ideation, NSSI and suicide attempt in univariate models. Suicide bereavement is likely associated with suicidal ideation through complicated grief [57] and suicide attempt via enhanced awareness of means, reduced fear of death or social modelling [58]. Considering the high levels of suicide bereavement in this occupational group (46.1%) and the greater probability of occupation drop-out among those bereaved by suicide compared to those bereaved by natural causes [59], it is clear that construction industry employers also need targeted approaches to support employees around suicide bereavement.

The only occupation-specific factor that was associated with suicidality outcomes was working 35–44 h per week which was associated with suicidal ideation and suicide attempt in the univariate models. It is likely that longer work hours impact on family and recreational activity time, thus decreasing wellbeing and increasing suicide risk [12]. However, working more than 45 h was not significantly associated with suicidality outcomes. Further research is needed to explore if the relationships between working hours and suicidality among construction workers is non-linear in other contexts. Financial worry was associated with all suicidality outcomes, but lower annual income was not. This may be due to the transient nature of the industry and perceived instability of future work rather than actual income [60] or, greater levels of financial worry might be associated with suicidality through greater levels of GAD. Interestingly, occupation skill level was not associated with any of the suicidality outcomes. This contrasts previous research that reported higher suicide rates among lower skilled construction occupation [61, 62]. As risk factors can vary across the suicidal spectrum and in relation to suicide mortality [8], ideation-to-action theories of suicide have focused on what risk factors, and in what combination, contribute to the development of suicidal ideation and the transition to a suicide attempt [63]. Therefore, sociodemographic factors might represent more distal risk factors (e.g. young age, not being in a relationship and suicide bereavement) that are associated with earlier suicidal trajectories, whereas occupation-specific factors such as low skill, low job control, job insecurity and financial stress might represent more proximal factors or acute stressors that trigger a more lethal suicide attempt and subsequent death by suicide among construction workers. Indeed, work stress and financial problems were more associated with individuals diagnosed with depression who died by suicide compared to those who attempted suicide [64]. Conversely, the fluid vulnerability theory emphasizes the temporal process of suicide risk fluctuation and the dynamic interactions of baseline risk factors, acute stressors, and protective factors over time [65]. Therefore, contextual factors relating to the current strong economic activity in the current Irish construction industry and the relatively high income and educational attainment among participants might have acted as protective factors and resulted in limited associations between occupation-specific factors and suicidality in this study. Indeed, wider social disadvantage and a disproportionate impact of the economic recession on job security and income have been implicated with regard to high suicide rates among lower skilled construction workers [61].

Depression and anxiety were the only risk factors retained in the multivariable analyses and were both associated with suicidal ideation, NSSI and suicide attempt. Those with severe depression had a 90% higher risk of suicidal ideation compared to those with no depression, 5.1 times the odds of NSSI and 18.8 times the odds of experiencing a suicide attempt. Similarly, those with severe anxiety had twice the risk of suicidal ideation compared to those with minimal anxiety, 4.7 times the odds of experiencing NSSI and 2.9 times the odds of experiencing a suicide attempt. These findings are consistent with previous studies that report a strong association between depression and suicidal ideation among male construction workers and apprentices [9, 26] and with suicide attempts among men more generally [55]. However, our findings regarding the association of anxiety with suicidal ideation contrasts from previous studies in the construction industry that reported no association [9]. This may be due to the difference in measures used to assess GAD between the studies - Tyler et al. asked participants to self-report if they had ever been treated for, or had symptoms of, anxiety in the past twelve months. Considering that men have lower mental health literacy and help-seeking behaviour, this may have resulted in undetected anxiety among male construction workers in their study. Nonetheless, the findings of this study may suggest that the symptoms or causes of depression and anxiety pose a similar threat to the development of suicidal ideation and NSSI, but the symptoms and causes of depression pose more of a threat to the development of a suicide attempt. However, due to the cross-sectional nature of this study, it is not possible to determine the causality or directionality of these mental health variables with the suicidality outcomes. Further research is needed to determine the ways in which depression and anxiety may exist as both a cause, and consequences of risk factors, for suicidal ideation, NSSI and suicide attempt. This study highlights that early identification, understanding and treatment of depression and anxiety among male construction workers has good scope to intervene in, and potentially prevent, suicidal trajectories among this occupational group. Considering that depression and anxiety are often undetected and under-reported among men, gender-sensitive screening instruments for the early detection of depressive and anxiety symptoms among male construction workers are recommended with appropriate referral pathways to professional treatment and/or further screening for suicidality based on severity of symptoms. Furthermore, existing suicide prevention interventions targeting male construction workers might benefit from widening their scope to include specific information on the signs and symptoms of depression and anxiety. Indeed, interventions that target men’s informal supports - promoting knowledge around men’s depression and anxiety symptoms and positive help-giving behaviours to upskill men’s friends and colleagues– have been highlighted as key in the identification, management and treatment of men’s depression and anxiety [66, 67].

This study has a number of limitations that should be taken into consideration. The cross-sectional nature of this study means we are unable to assess the directionality of the variables with the suicidality outcomes. There was a low response rate to the survey among contacted companies and a low participation rate among the estimated workforce across the 52 sites, so the sample may not be representative of the wider population. All participating companies were contractors who employed numerous other companies and subcontractors on-site. As the survey was carried out across the site and not exclusively with the staff of the participating companies, the true number of small/midsize enterprises and large enterprises in this study is unknown. As participants were self-selecting, individuals for whom suicide resonated more strongly, may have been more likely to take part, which could have introduced a non-response bias. Nonetheless, this is the first study of its kind and reports on a large sample size.

The study predominately assessed distal risk factors and did not include more proximal factors such as alcohol and drug misuse, psychosocial job adversity, physical working conditions or exposure to other chemical and biomechanical hazards which may have a more significant influence of some of these suicidality outcomes. Moreover, this study only conducted logistic regression analyses on the lifetime prevalence of the suicidality outcomes as numbers were too low to conduct reliable analyses on 12-month suicidality outcomes. Nonetheless, these limitations highlight the exploratory nature of this study, but the findings are still valuable for targeting specific subgroups of construction workers in need. Finally, the questions used to assess the suicidality outcomes were chosen to allow comparison with previous population studies. While these questions were taken from the revised Clinical Interview Schedule which has reported acceptable psychometric properties, the specific psychometric properties of the suicidality questions have not been reported in the literature.

## Conclusion

This is the first study to explore the prevalence and associated risk factors for suicidal ideation, NSSI and suicide attempt among male construction workers. This is an important first step if the construction industry is to develop more tailored, early intervention suicide prevention approaches to address a wider range of suicidal trajectories. The current study highlighted that younger age, working 35–44 h per week, not being in a relationship, suicide bereavement and financial worry were associated with different suicidality outcomes in the univariate models. Although they were not significant in the multivariate models, this study highlights the need for future early intervention programmes to consider these associations and for future research to account for these variables in order to create more conclusive evidence for the risk factors of suicidality among male construction workers. This study responded to recent calls for a better understanding of the relationship between mental health conditions and male suicide risk [68]. This study found that depression and anxiety is associated with suicidal ideation, NSSI and suicide attempts among male construction workers. Findings highlight a clear need for the early detection and treatment of depression and anxiety in order to intervene in, and potentially prevent, suicidality among this occupational group. More research is needed to explore the interaction and direction of depression and anxiety with suicidal ideation, NSSI and suicide attempt among male construction workers. Qualitative research is also needed to explore the gendered and lived experiences of depression and anxiety among male construction workers and how they, in turn, relate to different suicidal trajectories.

Finally, this is first study to explore the issue of suicidality among male construction workers in Ireland. In addition to providing important baseline data for the Irish construction industry, findings can inform a more targeted approach to ongoing suicide prevention efforts within the country. Although findings suggest that male construction workers have a lower prevalence of all suicidality outcomes compared to the general Irish population [38], suicidal ideation, NSSI and suicide attempt remains a significant issue among male construction workers that require specific attention. Longitudinal studies exploring suicidal ideation, NSSI and suicide attempt among male construction workers and males working in other occupations are recommended as well as the publication of robust data on the occupation of suicide decedents in Ireland.

### Electronic supplementary material

Below is the link to the electronic supplementary material.

Additional File 1

Additional File 2

## Data Availability

The datasets used and/or analyzed during the current study are available from the corresponding author on reasonable request.

## Abbreviations

CI: Confidence Interval
GAD: Generalized Anxiety Disorder
GB: Great Britain
PRR: Prevalence Rate Ratio
LRT: Likelihood Ratio Test
OR: Odds Ratio
PHQ: Patient Health Questionnaire
NSSI: Non-Suicidal Self-Injury
