# Supplementary material for: Prevalence and associated risk factors for suicidal ideation, non-suicidal self-injury and suicide attempt among male construction workers in Ireland
Source: BMC Public Health. 2024 May 8;24:1263. doi: 10.1186/s12889-024-18483-0 (PMC11077913; doi:10.1186/s12889-024-18483-0)
Supplement: Supplementary file 1 — Additional File 1 [file 12889_2024_18483_MOESM1_ESM.docx]

***Additional File 2: Participant Characteristics Overall***

|  | **N** | **%** |
| --- | --- | --- |
| **Age** | | |
| 18-29 | 597 | 38.1 |
| 30-49 | 762 | 48.6 |
| 50+ | 209 | 13.3 |
| **Region of birth** | | |
| Ireland | 1,274 | 81.0 |
| Europe & GB | 244 | 15.5 |
| Rest of world | 55 | 3.5 |
| **Sexual orientation** | | |
| Heterosexual | 1,535 | 97.9 |
| LGBTQ+ | 33 | 2.1 |
| **Relationship status** |  |  |
| In relationship | 1,134 | 71.7 |
| Not in relationship | 447 | 28.3 |
| **Living alone** | | |
| No | 1,428 | 90.7 |
| Yes | 147 | 9.3 |
| **Education** | | |
| Primary/Secondary | 579 | 36.7 |
| Trade/Dip | 703 | 44.5 |
| Tertiary | 298 | 18.9 |
| **Contract type** | | |
| Permanent | 1,059 | 67.5 |
| Not permanent | 511 | 32.6 |
| **Annual salary** | | |
| €29,999 or less | 336 | 21.8 |
| €30,000-€49,999 | 630 | 40.9 |
| €50,000-€69,999 | 398 | 25.8 |
| €70,000 or more | 177 | 11.5 |
| **Occupation** | | |
| Skilled Trade | 940 | 62.1 |
| Managerial | 276 | 18.2 |
| Unskilled Labour | 297 | 19.6 |
| **Shift work** | | |
| Always | 215 | 13.8 |
| Sometimes/seldom | 432 | 27.7 |
| Never | 913 | 58.5 |
| **Hours worked per week** | | |
| <35 hours worked per week | 364 | 23.1 |
| 35-44 hours worked per week | 951 | 60.3 |
| 45+ hours worked per week | 261 | 16.6 |
| **Commute time** | | |
| ≤1 hour | 993 | 62.9 |
| >1 hour | 585 | 37.1 |
| **Suicide bereavement** | | |
| No | 832 | 53.9 |
| Yes | 711 | 46.1 |
| **Financial worry** | | |
| Not at all worried | 606 | 38.4 |
| Somewhat worried | 302 | 19.1 |
| Worried | 385 | 24.4 |
| Very worried | 196 | 12.4 |
| Extremely worried | 90 | 5.7 |
| **GAD-7 category** | | |
| Minimal GAD | 1,002 | 63.6 |
| Mild GAD | 371 | 23.5 |
| Moderate GAD | 152 | 9.6 |
| Severe GAD | 51 | 3.2 |
| **PHQ-9 category** | | |
| No depression | 1,001 | 63.5 |
| Mild depression | 366 | 23.2 |
| Moderate depression | 139 | 8.8 |
| Moderate/severe depression | 52 | 3.3 |
| Severe depression | 18 | 1.1 |
