# Supplementary material for: Prevalence and associated risk factors for suicidal ideation, non-suicidal self-injury and suicide attempt among male construction workers in Ireland
Source: BMC Public Health. 2024 May 8;24:1263. doi: 10.1186/s12889-024-18483-0 (PMC11077913; doi:10.1186/s12889-024-18483-0)
Supplement: Supplementary file 2 — Additional File 2 [file 12889_2024_18483_MOESM2_ESM.docx]

*Additional File 1: Survey Questions.*

**Q1. What is your age in years?**

**Q2. What is your country of birth?**

**Q3. What is your sexual orientation?**

☐Straight

☐Gay

☐Bisexual

☐Other

**Q4. What category best describes your current relationship status?**

☐Single, not in a committed relationship

☐Single, in a committed relationship

☐Married

☐Separated or divorced

☐Widowed

**Q5. Do you live alone?**

**(give the answer that best describes your living arrangements most of the week)**

☐Yes

☐No

**Q6. On average, how long is your commute to work each day?**

**(the total time it takes to go both ways)**

☐ Less than 30 minutes

☐ 30 minutes - 1 hour

☐ 1 – 2 hours

☐ 2 – 3 hours

☐ 3 – 4 hours

☐ More than 4 hours

**Q7. Which of the following have you completed to date?**

**(select more than one if applicable)**

☐ No formal education

☐ Primary school education

☐ Secondary school education

☐ Trade qualification or diploma

☐ Undergraduate degree

☐ Graduate degree (masters or PhD)

**Q8. What is your specific trade or occupation?**

☐Electrical trade

☐Metal trade

☐Plumbing trade

☐Structural trade

☐Finishing trade

☐Civil and outdoor labour

☐Safety/quality/planning

☐Project management

☐Admin

☐Other (please specify): **___________________________**

**Q9. What is your type of work contract?**

☐Permanent contract

☐Temporary contract

☐Subcontract

☐Agency contract

☐None of the above

☐Other type of contract (please specify): _____________________________

**Q10. What are your average earnings over one year before tax?**

**(e.g. weekly income x52 or monthly income x12)**

☐Less that €20,000

☐€20,000 to €29,999

☐€30,000 to €39,999

☐€40,000 to €49,999

☐€50,000 to €59,999

☐€60,000 to €69,999

☐More than €70,000

**Q11. Do you do shift work?**

☐Always

☐Sometimes

☐Never

**Q12. How many hours per week do you work on average?**

☐ Less than 35 hours

☐ 35-39 hours

☐ 40 – 44 hours

☐ 45 – 49 hours

☐ 50 – 54 hours

☐ 55 – 59 hours

☐ 60+ hours

**Q13. To what extent do you worry about finances?**

☐ 1=not worried at all

☐ 2

☐ 3

☐ 4

☐ 5 = neutral

☐ 6

☐ 7

☐ 8

☐ 9

☐ 10= extremely worried

| **Q14. Over the last 2 weeks, how often have you been bothered by the following problems:** | | | | |
| --- | --- | --- | --- | --- |
| (Use “✔” to indicate your answer”) | **Not at all** | **Several days** | **More than half the days** | **Nearly every day** |
| 1. Feeling nervous, anxious or on edge |  |  |  |  |
| 1. Not being able to stop or control worrying |  |  |  |  |
| 1. Worrying too much about different things |  |  |  |  |
| 1. Trouble relaxing |  |  |  |  |
| 1. Being so restless that it is hard to sit still |  |  |  |  |
| 1. Becoming easily annoyed or irritable |  |  |  |  |
| 1. Feeling afraid as if something awful might happen |  |  |  |  |

| **Q15. Over the last 2 weeks, how often have you been bothered by the following problems:** | | | | |
| --- | --- | --- | --- | --- |
| (Use “✔” to indicate your answer”) | **Not at all** | **Several days** | **More than half the days** | **Nearly every day** |
| 1. Little interest or pleasure in doing things |  |  |  |  |
| 1. Feeling down, depressed or hopeless |  |  |  |  |
| 1. Trouble falling or staying asleep, or sleeping too much |  |  |  |  |
| 1. Feeling tired or having little energy |  |  |  |  |
| 1. Poor appetite or overeating |  |  |  |  |
| 1. Feeling bad about yourself – or that you are a failure or have let yourself or your family down |  |  |  |  |
| 1. Trouble concentrating on things, such as reading the newspaper or watching TV |  |  |  |  |
| 1. Moving or speaking so slowly that other people could have noticed? Or the opposite — being so fidgety or restless that you have been moving around a lot more than usual |  |  |  |  |
| 1. Thoughts that you would be better off dead or of hurting yourself in some way |  |  |  |  |

**Q16. There may be times in everyone’s life when they become miserable and depressed and**

**may feel like taking drastic action because of these feelings. Have you ever thought of harming yourself or taking your life, even if you would not do it?**

☐ Yes

☐ No

**If you answered yes to the previous question, when did this most recently occur?**

☐ In the past year

☐ More than one year ago

**Q17. Have you ever deliberately harmed yourself in any way but not with the intention of taking your own life?**

☐Yes

☐No

**If you answered yes to the previous question, when did this most recently occur?**

☐ In the past year

☐ More than one year ago

**Q18. Have you ever made an attempt to take your own life?**

• ☐Yes

• ☐No

**If you answered yes to the previous question, when did this most recently occur?**

• ☐ In the past year

• ☐ More than one year ago

**Q19. Did someone close to you (i.e. family, relative, friend, colleague, neighbour) die by suicide?**

• ☐Yes

• ☐No
